# Health insurance as a moderator in the relationship between financial toxicity and medical cost‐coping behaviors: Evidence from patients with lung cancer in China

**DOI:** 10.1002/cam4.6911

**Published:** 2024-01-03

**Authors:** Yongchun Cui, Jingjing Lv, Xiaoyu Hu, Dawei Zhu

**Affiliations:** ^1^ Shandong Cancer Hospital and Institute Shandong First Medical University and Shandong Academy of Medical Sciences Jinan China; ^2^ Expanded Program Immunization Division of Shandong Provincial Center for Disease Control and Prevention Shandong Provincial Key Laboratory of Infectious Disease Control and Prevention Jinan China; ^3^ School of Public Health, Cheeloo College of Medicine Shandong University Jinan China; ^4^ China Center for Health Development Studies Peking University Beijing China

**Keywords:** China, financial toxicity, health insurance, medical cost‐coping behaviors, patients with lung cancer

## Abstract

**Objective:**

This study investigates the relationship between financial toxicity and medical cost‐coping behaviors (MCCB) in Chinese patients with lung cancer, with a particular focus on the moderating role of health insurance.

**Methods:**

We surveyed 218 patients with lung cancer and assessed their Comprehensive Score for Financial Toxicity (COST) and self‐reported MCCB. Patients were categorized into Urban Employee's Basic Medical Insurance (UEBMI) group and Urban–Rural Resident Basic Medical Insurance Scheme (URRBMI) groups by their medical insurance, and matched for socioeconomic, demographic, and disease characteristics via propensity score.

**Results:**

Significant different characteristics were noted between UEBMI patients and URRBMI patients. Patients with UEBMI had higher COST scores but lower levels of MCCB compared to URRBMI patients in the original dataset. After data matching, multivariate logit regression analysis showed that better financial toxicity was associated with lower levels of MCCB (OR = 0.95, 95% CI: 0.92–0.99). Health insurance type did not have a direct association with cost‐coping behaviors, but an interaction was observed between health insurance type and financial toxicity. Among patients with URRBMI, better financial toxicity was associated with lower levels of cost‐coping behaviors (OR = 0.89, 95% CI: 0.83–0.95). Patients with UEBMI had a lower probability of engaging in any cost‐coping behaviors in situations of worse financial toxicity compared to patients with URRBMI.

**Conclusion:**

The findings suggest that financial toxicity is correlated with MCCB in Chinese patients with lung cancer. The type of health insurance, specifically UEBMI and URRBMI, plays a moderating role in this relationship. Understanding these dynamics is essential for developing targeted interventions and policies to mitigate financial toxicity and improve patients' management of medical costs.

## 1 INTRODUCTION

Lung cancer presents a major health challenge in China, characterized by high morbidity and mortality rates.^1^ Trachea, bronchus, and lung cancer (referred to as TBL) incurs the highest economic cost among all types of cancers, totaling 3.9 trillion US dollars.^2^ The incidence of lung cancer has risen over the past decade,^3^ increasing the physical, psychological, and economic strain on patients. Hospitalization costs constitute a significant part of the overall medical expenses for cancer in China.^4^, ^5^, ^6^, ^7^ This financial strain, often compounded by a loss of income, can adversely affect patients, resulting depression, and diminished sense of financial well‐being. Zafar et al. introduced the term “financial toxicity” to describe these adverse impacts.^8^ Financial toxicity refers to the objective economic burden and subjective economic distress experienced by cancer patients due to high medical costs, including insurance premiums, co‐payments, deductibles, and prescription drugs.^9^

The detrimental effects of financial toxicity go beyond the immediate economic burden. de Souza et al. developed the Comprehensive Score for Financial Toxicity (COST) and demonstrated a strong association between higher levels of financial toxicity and diminished health‐related quality of life in cancer patients in their study.^10^ In terms of medical cost‐coping behaviors (MCCB), Zafar (2016) emphasized the negative outcome of financial toxicity, such as medication nonadherence.^11^ Cancer patients who face significant financial burdens may struggle to afford their medications, leading to suboptimal treatment outcomes. Similarly, delaying treatment, reducing medication doses, and discontinuing treatment have been identified as coping strategies employed by patients to manage financial constraints.^9^

Health insurance coverage is a pivotal factor impacting the relationship between financial toxicity and MCCB. Health insurance affects access to care, coverage of treatments, and out‐of‐pocket (OOP) expenses. Zullig et al. (2013) found that inadequate health insurance coverage was associated with higher engagement in cost‐coping strategies and lower adherence to prescription medications among cancer patients.^12^ Similarly, Dusetzina et al. (2014) observed that higher OOP costs under insurance plans was associated with lower adherence to tyrosine kinase inhibitors.^13^ These findings highlight the importance of health insurance in enabling patients to navigate the financial obstacles posed by cancer treatment.

The Urban Employee's Basic Medical Insurance (UEBMI) and the Urban–Rural Resident Basic Medical Insurance Scheme (URRBMI) cover over 90% of the population in China. UEBMI primarily covers urban residents in the formal sector and offers higher financing and more generous benefits, whereas URRBMI is designed for urban and rural residents who are not included in UEBMI, providing lower premiums and reimbursement rates. This includes individuals such as students, children, the elderly, and informal workers. For patients with lung cancer, the share of OOP cost was roughly 54% and 74% in URRBMI and UEBMI, respectively.^5^ Health insurance status has been associated with differences in outcomes and disparities in treatment in patients with lung cancer. Ellis et al has shown that uninsured patients or those with lower reimbursement health insurance have significantly lower survival rates compared to patients with high‐reimbursement health insurance.^14^ Wang et al have confirmed that NSCLC patients with good insurance status have better survival rates. Patient with non‐small cell lung cancer who hold UEBMI or Free Medical Care (FMC) have 5‐year survival rates of 23.38% and 14.76%, respectively. There are also differences in the 10‐year survival rates, which are 12.27% and 10.25%, respectively.^15^ However, the role of insurance disparities in the correlation between financial toxicity and MCCB is less known and The variations in OOP expenses highlight the need to investigate how insurance differences impact financial toxicity and MCCB.

Understanding the correlation between financial toxicity and MCCB is crucial for developing strategies to mitigate the adverse effects of financial distress on cancer patients. Additionally, the role of health insurance as a moderator within this relationship needs to be explored. The aim of this study is to explore the association between financial toxicity and MCCB in patients with lung cancer, emphasizing the moderating role of health insurance. The ultimate goal is to develop effective interventions and support systems that alleviate the detrimental impacts of financial toxicity on cancer treatment and patient care.

## 2 METHODS

### 2.1 Setting, participants, and recruitment

An anonymous, cross‐sectional survey study was conducted. Eligible participants were pathologically diagnosed with American Joint Committee on Cancer (8th edition) stage I to IV lung cancer, receiving tumor treatment for 2 months or longer, 18 years or older, and able to read and write in Chinese. The tumor treatment included neoadjuvant therapy, surgery, radiation therapy, and chemotherapy. A total of 251 patients with lung cancer were initially approached in Shandong Cancer Hospital and Institute (a tertiary academic medical center in the eastern of China) during March to May 2021. Of the 251 approached, 246 (98.0%) patients consented to participate, with 218 providing valid questionnaires after excluding 24 invalid questionnaires and four patients without insurance. This study was approved by the Ethics Committee of the Affiliated Cancer Hospital of Shandong First Medical University (No. SDTHEC2021002001).

### 2.2 Variables and measures

Financial toxicity was assessed using COST, a proven method of measuring financial toxicity in China and other countries.^8^, ^9^ The scale consisted of 11 items relating to patients' subjective perceptions with in the past 7 days, rated a 5‐point Likert scale: (0) “Not at all”, (1) “A little bit”, (2) “Somewhat”, (3) “Quite a bit”, and (4) “A lot”. The final cumulative score (0–44) was obtained, with lower scores indicated higher financial toxicity.^10^

Cost‐coping behaviors were modeled after questions from the Medical Expenditure Panel Survey and drawn from prior literature.^16^, ^17^ Participants selected “yes” if, in the past year,they had engaged in any 1 of 5 cost‐coping behaviors because they were worried about the cost: “taking a smaller dose/fewer pill of a medication than prescribed”, “not filling a prescription”, “refuse recommended medical test or treatment”, “decreasing the number of treatments”, and “delaying or abandoning treatment”. These questions have been widely employed in numerous research studies to assess MCCB.^12^, ^18^, ^19^, ^20^, ^21^, ^22^, ^23^

We also collected patients' sociodemographic information including insurance (URRBMI and UEBMI), residence type (urban and rural), monthly family income, education level (those with junior school education or less and those with a high school education or above), and household savings (the total sum of savings within patients' households, which encompasses not only traditional savings accounts but also electronic currency, such as WeChat and Alipay, as well as cash flow). Disease information was extracted from the hospital's electronic medical records which included details on the patients' cancer stage, pathological diagnosis, and the number of chemotherapy cycles.

### 2.3 Statistical analysis

Descriptive statistics were used to summarize patient characteristics by insurance type. Pearson's chi‐square tests and *t* test were used to compare the difference between two insurance groups. Since two health insurance schemes cover different segments of the population, the differences in patient characteristics may lead to biased estimates using the original data. In order to decrease this potential bias, we constructed a matched dataset of patients with lung cancer to analyze the correlation between financial toxicity and MCCB. These patients were matched 1:1 by their propensity score (PS) using the greedy matching algorithm. We derived the PS from a multi‐logistical regression model with the variables age, sex, residence type, income, education, household savings, cancer stage, pathological diagnosis, and chemotherapy. We utilized multinomial logistic regression to examine the associations between financial toxicity and MCCB. To further investigate the role of insurance type as a potential moderator in this relationship, we incorporated an interaction term between insurance type and COST. Our analysis involved constructing three distinct models, referred to as Models 1, 2, and 3. Model 1 focus on the relationship between MCCB and COST score, adjusted for various covariates, including age, sex, residence type, family income, education, household savings, cancer stage, pathological diagnosis, and chemotherapy cycle. Model 2 extends Model 1 by including health insurance in the analysis. Model 3 further include the interaction between financial toxicity and insurance type.

All statistical analyses were conducted with Stata/SE 17.0 software (Stata Corp LP, College Station, TX, USA). A two‐tailed *p* value of <0.05 was considered statistically significant.

## 3 RESULTS

As shown in Table 1, among the 218 patients with lung cancer, 147 were covered by URRBMI, the mean age was 59.7, 64.7% were male, and 62.8% were rural residents. There are significant differences in age, residence type, monthly income, education level, and household saving between patients with different types of insurance. URRBMI patients were slightly older (mean age 60.7 vs. 57.6 years for UEBMI, *p* = 0.022). The residence type showed a substantial contrast, with 80.3% of URRBMI patients in rural areas compared to 26.8% of UEBMI patients (*p* < 0.001). UEBMI patients had higher monthly family income (8.7 vs. 7.9 for URRBMI, *p* = 0.005). Education level also differed significantly, with 50.3% of URRBMI patients having junior school education or less, while 90.1% of UEBMI patients had a high school education or above (p < 0.001). Household savings varied, with 60.5% of URRBMI patients having 0–50,000 units, and 60.6% of UEBMI patients having 50,000 units or more (*p* = 0.003). One hundred and thirty four patients had cancer stage IV, 55.5% were adenocarcinoma, and 36.2% received more than 6 cycles of chemotherapy.

**TABLE 1:** Demographics and Clinical Information of the Patients.

	Total (*N* = 218)	URRBMI (*N* = 147)	UEBMI (*N* = 71)	*p* Value
Age, Mean (SD)	59.7 (9.6)	60.7 (9.0)	57.6 (10.5)	0.022
Sex, *N* (%)				0.236
Female	77 (35.3)	48 (32.7)	29 (40.8)	
Male	141 (64.7)	99 (67.3)	42 (59.2)	
Residence type, *N* (%)				<0.001
Urban	81 (37.2)	29 (19.7)	52 (73.2)	
Rural	137 (62.8)	118 (80.3)	19 (26.8)	
Monthly family income (CNY, log), Mean (SD)	8.2 (1.9)	7.9 (2.2)	8.7 (1.2)	0.005
Education, *N* (%)				<0.001
Junior school or less	81 (37.2)	74 (50.3)	7 (9.9)	
High school or above	137 (62.8)	73 (49.7)	64 (90.1)	
Household savings, *N* (%)				0.003
0–50,000	117 (53.7)	89 (60.5)	28 (39.4)	
50,000 +	101 (46.3)	58 (39.5)	43 (60.6)	
Cancer stage, *N* (%)				0.319
I‐III	84 (38.5)	60 (40.8)	24 (33.8)	
IV	134 (61.5)	87 (59.2)	47 (66.2)	
Pathological diagnosis, *N* (%)				0.410
Adenocarcinoma	121 (55.5)	77 (52.4)	44 (62.0)	
Small cell carcinoma	54 (24.8)	39 (26.5)	15 (21.1)	
Others	43 (19.7)	31 (21.1)	12 (16.9)	
Chemotherapy cycle, *N* (%)				0.935
1–6	139 (63.8)	94 (63.9)	45 (63.4)	
6+	79 (36.2)	53 (36.1)	26 (36.6)	

Abbreviations: CNY log, monthly family income; CNY, monthly family income in Chinese Yuan transformed into logarithmic units; SD, standard deviations; URRBMI, urban–rural resident basic medical insurance; UEBMI, urban employee's basic medical insurance.

As shown in Table 2, patients with UEBMI have higher COST with lower MCCB than patients with URRBMI in original data set. Table S1 shown that, there is no statistically significant difference between URRBMI and UEBMI patients after the matching. Figure S1 shown Kernel density of PS by treatment status before and after matching. In the matched data, the difference in COST and MCCB were insignificant.

**TABLE 2:** Comprehensive score for financial toxicity and medical cost‐coping behaviors.

	Unmatched			Matched		
	URRBMI (*N* = 147)	UEBMI (*N* = 71)	*p* Value	URRBMI (*N* = 69)	UEBMI (*N* = 69)	*p* Value
COST, Mean (SD)	13.39(8.73)	19.51(10.50)	<0.001	19.04(10.18)	19.97(10.48)	0.511
MCCB, *N* (%)	40(21.56)	7(9.86)	0.004	11(10.14)	16(14.54)	0.430

Abbreviations: COST, comprehensive score for financial toxicity; MCCB, medical cost‐coping behaviors; SD, standard deviations; URRBMI, urban–rural resident basic medical insurance; UEBMI, urban employee's basic medical insurance.

Table 3 presents the OR and 95% CI of multivariate logit regression for MCCB. Model 1 provides evidence regarding whether financial toxicity leads to increased levels of MCCB. In Model 1, having better financial toxicity (higher COST score) is significantly associated with lower levels of MCCB (OR = 0.95, 95% CI: 0.92–0.99). Health insurance type is not associated with MCCB in Model 2. Model 3 shown that health insurance type interacts with financial toxicity to influence MCCB. In Model 3, better financial toxicity (higher COST score) is significantly associated with lower levels of MCCB among patients with URRBMI (OR = 0.89, 95% CI: 0.83–0.95), patients with UEBMI have a lower probability of any MCCB in the situation of worse financial toxicity compared to patients with URRBMI, and UEBMI lessens the burden of financial toxicity.

**TABLE 3:** Multivariate logit regression for medical cost‐coping behaviors, OR (95%CI).

	Unmatched			Matched		
	Model 1	Model 2	Model 3	Model 1	Model 2	Model 3
COST	0.95* (0.92, 0.99)	0.96* (0.92, 1.00)	0.95* (0.91, 1.00)	0.94** (0.89, 0.98)	0.94** (0.89, 0.98)	0.89*** (0.83, 0.95)
UEBMI		0.52 (0.18, 1.47)	0.37 (0.06, 2.16)		0.59 (0.21, 1.62)	0.11** (0.02, 0.55)
COST* UEBMI			1.02 (0.93, 1.12)			1.12** (1.03, 1.22)

*Note*: All models were adjusted for age, sex, residence type, family income, education, household savings, cancer stage, pathological diagnosis, and chemotherapy cycle; *p < 0.05, **p < 0.01, ***p < 0.001.

Abbreviations: COST, comprehensive score for financial toxicity; URRBMI, urban–rural resident basic medical insurance; UEBMI, urban employee's basic medical insurance.

**FIGURE 1:**
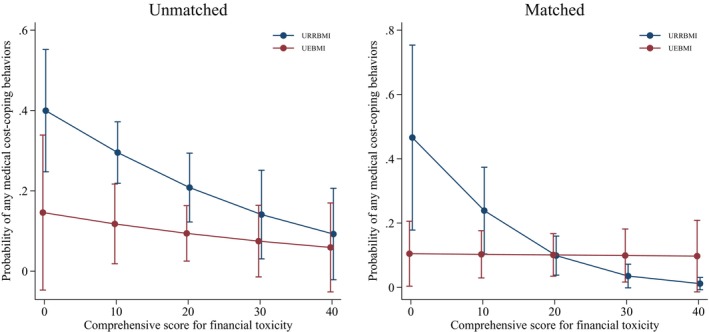
Associations between comprehensive score for financial toxicity with medical cost‐coping behaviors by insurance type. For patients with UEBMI, worse financial toxicity (lower COST score) results in higher probability of any medical cost‐coping behaviors. For patients with URRBMI, financial toxicity does not appear to influence medical cost‐coping behaviors significantly.

Figure 1 provides a graphical representation of the interaction term. For patients with UEBMI, worse financial toxicity (lower COST score) results in a higher probability of any MCCB. For patients with URRBMI, financial toxicity does not appear to influence medical cost‐coping behavior significantly. Thus, UEBMI is protective for patients with worse financial toxicity.

## 4 DISCUSSION

In China, the increasing emphasis on early screening and diagnosis has led to a trend of lung cancer being detected at younger ages. Our study aimed to examine the correlation between financial toxicity and MCCB among patients with lung cancer, with a specific focus on the moderating role of health insurance. The analysis revealed that patients with URRBMI had higher levels of financial toxicity, indicated by lower COST scores, and exhibited higher levels of MCCB compared to patients with UEBMI. As financial toxicity increases, patients are more likely to engage in MCCB, and UEBMI acts as a protective factor for patients facing worse financial toxicity.

Our study found a significant positive correlation between financial toxicity and MCCB among patients with lung cancer in China. The findings of our study align with previous research conducted in China and other countries, highlighting the adverse effects of financial toxicity on patients' treatment decisions and healthcare utilization.^10^, ^13^ The burden of high treatment costs exacerbates financial distress, leading to various coping strategies employed by patients. Nonadherence to prescribed medication, prescription abandonment, refusal of recommended treatment options, and reduction in clinic visits are commonly observed cost‐coping behaviors.

Hospitalization expenses constitute a significant portion of the total medical and health expenditure on cancer in China.^24^, ^25^, ^26^ The financial burden of lung cancer treatment can be particularly overwhelming for patients and their families. Financial toxicity can have detrimental consequences for patients with lung cancer in China, including increased psychological distress, decreased treatment adherence, and decreased satisfaction with their financial situations. These negative effects can further compromise patients' overall well‐being and quality of life, hindering their ability to cope with the physical and emotional challenges of the disease.^7^, ^20^, ^27^

Notably, this study also investigated the moderating role of health insurance coverage in the relationship between financial toxicity and MCCB. The results revealed that adequate health insurance coverage acted as a protective factor, buffering the negative impact of financial toxicity on patients' coping strategies. Patients with lung cancer with comprehensive health insurance were less likely to engage in cost‐coping behaviors and demonstrated higher levels of treatment adherence. These results are consistent with previous studies that have shown the beneficial effects of health insurance coverage on treatment adherence and healthcare utilization.^12^

UEBMI and URRBMI aim to provide financial protection and improve healthcare access in China. However, coverage gaps and reimbursement policy variations persist, leaving certain patients susceptible to financial toxicity.^28^, ^29^, ^30^ UEBMI offers comprehensive coverage, including higher reimbursement rates for hospitalization, specialized treatments, and expensive medications, reducing the need for cost‐coping behaviors. Conversely, URRBMI provides more limited coverage, with lower reimbursement rates and a narrower range of services, leading to higher OOP expenses and increased financial distress. UEBMI beneficiaries generally have stable employment and higher incomes, enhancing affordability and reducing financial toxicity. In contrast, URRBMI targets a broader population, including those with lower socioeconomic status and rural residents, who may struggle with premiums or copayments, elevating the risk of financial toxicity and hindering their ability to cope with medical costs effectively.

Our study has important implications for healthcare providers, policymakers, and patients with lung cancer. Firstly, it highlights the significance of early identification and assessment of financial toxicity. Integrating screening tools and interventions for financial distress into routine clinical practice can help identify at‐risk patients and provide appropriate support and resources. Secondly, our findings highlight the need for comprehensive health insurance policies that adequately cover cancer treatments and associated costs. Policymakers should consider implementing measures to enhance health insurance coverage, reduce OOP expenses, and minimize financial barriers to accessing necessary care. Collaborative efforts among healthcare institutions, insurance providers, and policymakers are crucial to ensure that cancer patients receive optimal care without experiencing excessive financial burdens. Thirdly, healthcare providers have a vital role in supporting patients in navigating the financial challenges of their treatment journey.^31^ Patient education and counseling on available financial assistance programs, cost‐saving strategies, and coping mechanisms can empower patients to make informed decisions and alleviate their financial burden.^9^ Additionally, understanding the differences between UEBMI and URRBMI and their impact on financial toxicity and cost‐coping behaviors is essential. This knowledge can aid in developing targeted interventions and policies that address the unique challenges faced by patients in different insurance schemes. By improving access to affordable healthcare and reducing financial burdens, we can enhance the overall well‐being and treatment outcomes of patients with lung cancer.

While this study provides valuable insights into the correlation between financial toxicity, MCCB, and the moderating role of health insurance, several limitations should be noted. Firstly, the study focused specifically on patients with lung cancer, and the findings may not be generalized to other cancer types. Additionally, our sample from a specialized Cancer Hospital in eastern China, which may attract complex cases, may not fully represent all patients with lung cancer. Future research should explore these relationships in diverse cancer populations to enhance the understanding of financial toxicity in various contexts. Secondly, the study relied on self‐report measures, which may be subject to recall and reporting biases. Thirdly, analyzing each of the five types of MCCB separately was not feasible due to the small sample size and the rarity of these behaviors, resulting in insufficient statistical power. Future studies could incorporate objective measures of treatment adherence and healthcare utilization to provide a more comprehensive assessment of patients' behaviors. Furthermore, the association between radiotherapy and economic toxicity has been confirmed in other cancer types, such as breast cancer.^32^ However, there is currently a research gap concerning the relationship between radiation therapy and the financial toxicity experienced by patients with lung cancer. This highlights an important and worthwhile area for future investigation.

## 5 CONCLUSION

In summary, our study provides insights into the relationship between financial toxicity and MCCB among patients with lung cancer. The findings suggest that addressing financial toxicity is crucial to mitigate the negative impact on patients' ability to cope with medical costs. Health insurance, particularly UEBMI, appears to play a protective role in alleviating the burden of financial toxicity. These findings underscore the importance of implementing interventions and policies aimed at reducing financial toxicity and improving access to affordable cancer care.

## AUTHOR CONTRIBUTIONS

**Yongchun Cui:** Conceptualization (lead); data curation (lead); formal analysis (equal); funding acquisition (equal); investigation (supporting); methodology (supporting). **Jingjing Lv:** Software (supporting); supervision (supporting); validation (equal); visualization (equal); writing – original draft (lead); writing – review and editing (lead). **Xiaoyu hu:** Data curation (lead); investigation (lead); validation (equal); visualization (equal); writing – original draft (supporting); writing – review and editing (supporting). **Dawei Zhu:** Project administration (lead); resources (lead); software (equal); supervision (equal); validation (supporting); visualization (supporting).

## FUNDING INFORMATION

National Institute of Hospital Administration, National Health Commission of the PRC (YLZLXZ23G016).

## CONFLICT OF INTEREST STATEMENT

The authors declare no conflict of interest.

## ETHICS STATEMENT

This study was approved by the Ethics Committee of the Affiliated Cancer Hospital of Shandong First Medical University (No. SDTHEC2021002001).

## CONSENT

Informed consent was obtained from all participants included in the study.

## Supporting information


Figure S1.

Table S1.

## Data Availability

The data that support the findings of this study are available upon request, but restrictions apply to the availability of these data.
