# Supplementary figures and images for: Health insurance as a moderator in the relationship between financial toxicity and medical cost‐coping behaviors: Evidence from patients with lung cancer in China
Source: Cancer Med. 2024 Jan 3;13(1):e6911. doi: 10.1002/cam4.6911 (PMC10807627; doi:10.1002/cam4.6911)

**
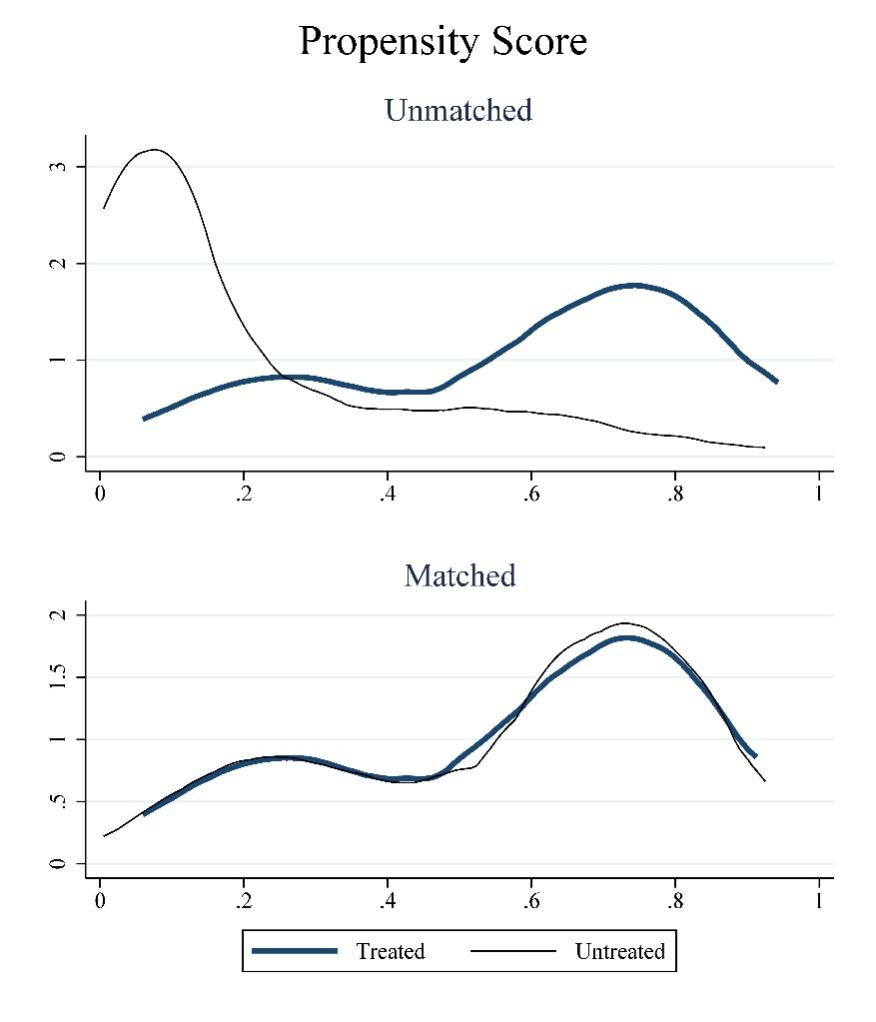
**

**FIGURE S1.** **Kernel density of propensity scores by treatment status before and after matching.**

Supplement: Supplementary file 1 — Figure S1. [file CAM4-13-e6911-s001.docx]
