# Supplementary material for: Health insurance as a moderator in the relationship between financial toxicity and medical cost‐coping behaviors: Evidence from patients with lung cancer in China
Source: Cancer Med. 2024 Jan 3;13(1):e6911. doi: 10.1002/cam4.6911 (PMC10807627; doi:10.1002/cam4.6911)
Supplement: Supplementary file 2 — Table S1. [file CAM4-13-e6911-s002.docx]

**Appendix**

**TABLE S1 Balancing test estimates for matching**

| Variable | Mean | | T-test | |
| --- | --- | --- | --- | --- |
|  | Treated | Control | T-stat. | P-value |
|  |  |  |  |  |
| Age | 57.841 | 58.194 | -0.22 | 0.830 |
| Male | .6087 | .73001 | -1.52 | 0.132 |
| Rural | .27536 | .29559 | -0.26 | 0.794 |
| High school or above | .89855 | .93765 | -0.83 | 0.406 |
| Income (log) | 8.7065 | 8.928 | -1.06 | 0.292 |
| Household savings 50 000 + | .5942 | .62956 | -0.42 | 0.673 |
| Cancer stage IV | .65217 | .71327 | -0.77 | 0.444 |
| Pathological diagnosis small cell carcinoma | .21739 | .1757 | 0.61 | 0.541 |
| Pathological diagnosis Others | .17391 | .17873 | -0.07 | 0.941 |
| Chemotherapy cycle 6+ | .36232 | .37028 | -0.10 | 0.923 |
